# HIV-Exposed Uninfected Infants Show Robust Memory B-Cell Responses in Spite of a Delayed Accumulation of Memory B Cells: an Observational Study in the First 2 Years of Life

**DOI:** 10.1128/CVI.00149-16

**Published:** 2016-07-05

**Authors:** Eunice W. Nduati, Irene N. Nkumama, Faith K. Gambo, Daniel M. Muema, Miguel G. Knight, Amin S. Hassan, Margaret N. Jahangir, Timothy J. Etyang, James A. Berkley, Britta C. Urban

**Affiliations:** a Kenya Medical Research Institute/Wellcome Trust Research Programme, Centre for Geographic Medicine Research Coast, Kilifi, Kenya; b Nuffield Department of Clinical Medicine, University of Oxford, Oxford, United Kingdom; c Kilifi County Hospital, Kilifi, Kenya; d Liverpool School of Tropical Medicine, Liverpool University, Liverpool, United Kingdom; Duke University Medical Center

## Abstract

Improved HIV care has led to an increase in the number of HIV-exposed uninfected (HEU) infants born to HIV-infected women. Although they are uninfected, these infants experience increased morbidity and mortality. One explanation may be that their developing immune system is altered by HIV exposure, predisposing them to increased postnatal infections. We explored the impact of HIV exposure on the B-cell compartment by determining the B-cell subset distribution, the frequency of common vaccine antigen-specific memory B cells (MBCs), and the levels of antibodies to the respective antigens in HEU and HIV-unexposed uninfected (HUU) infants born to uninfected mothers, using flow cytometry, a B-cell enzyme-linked immunosorbent spot assay, and an enzyme-linked immunosorbent assay, respectively, during the first 2 years of life. For the majority of the B-cell subsets, there were no differences between HEU and HUU infants. However, HIV exposure was associated with a lower proportion of B cells in general and MBCs in particular, largely due to a lower proportion of unswitched memory B cells. This reduction was maintained even after correcting for age. These phenotypic differences in the MBC compartment did not affect the ability of HEU infants to generate recall responses to previously encountered antigens or reduce the antigen-specific antibody levels at 18 months of life. Although HIV exposure was associated with a transient reduction in the proportion of MBCs, we found that the ability of HEU infants to mount robust MBC and serological responses was unaffected.

## INTRODUCTION

The use of highly active antiretroviral therapy (HAART), improved obstetric management, and formula feeding have reduced vertical HIV infection to almost zero in the developed countries (1), with some progress being made in resource-poor countries (2). Consequently, the number of HIV-exposed uninfected (HEU) infants born to HIV-infected women has and will continue to increase, particularly in regions where HIV infection in women of childbearing age is still prevalent (3).

Increased rates of morbidity and mortality are reported in HEU infants (4–8). While these may be partly explained by increased exposure to environmental antigens and poor maternal health, it is possible that *in utero* exposure to HIV antigens, antiretroviral drugs, and an altered placental cytokine environment may affect the developing immune system, predisposing HEU infants to increased postnatal infections.

The impact of maternal chronic infection on fetal immunomodulation and, specifically, of HIV exposure has previously been reviewed (9–11). For HIV, exposure in infants has been associated with an activated intrauterine immune environment (12, 13) and reduced T-cell counts and polyfunctionality (14–17). While the available evidence has largely focused on the potential disruptions to the T-cell compartment in HEU infants, much less is known about the impact of HIV exposure on the B-cell compartment, with a majority of the studies concentrating on serological parameters (10). Previous observations of the profound effect of HIV infection on B cells and their function (18, 19) may extend, albeit subtly, to HIV exposure in the absence of infection. Elevated levels of total immunoglobulin in HEU infants compared to those in HIV-unexposed uninfected (HUU) infants born to HIV-uninfected mothers have been reported to persist for more than 2 years (20). When specific antibody responses against childhood immunizations were measured, HEU infants responded with antibody levels similar to those in HUU infants (21–23). However, other studies have reported a larger proportion of nonresponders to hepatitis B vaccine (24), diminished neutralizing antibodies to poliovirus vaccine (25), lower antibody avidity (23), and reduced opsonization for some of the pneumococcal polysaccharides of conjugate vaccine (26) among HEU infants. Of the few studies that have investigated the impact of HIV exposure on B cells, one reported increased B-cell apoptosis in HEU infants (27), whereas others observed a higher percentage of CD19^+^ cells (16, 28). Recently, similar proportions of B-cell subsets were reported in HEU infants and HUU infants at age 6, 12, and 18 months (29).

Previous studies have, however, not associated observed phenotypes with B-cell function. In the current study, we sought to investigate the impact of maternal HIV infection on the infant's developing B-cell compartment during the first 2 years of life by determining the phenotypic composition of the B-cell compartment and associating this with the induction and maintenance of antigen-specific memory B cells and antibodies in response to common childhood vaccines in HEU and HUU infants.

## MATERIALS AND METHODS

### Study population and recruitment.

The study was conducted at the Comprehensive Care and Research Clinic (CCRC), Kilifi County Hospital (KCH), prior to the 2012 national integration of prevention of mother-to-child transmission of HIV (PMTCT) services with mother-to-child health (MCH) services. PMTCT care and testing were provided per Kenyan guidelines (30) and as previously reported (31). In summary, the guidelines recommended that all pregnant women be tested for HIV during their first antenatal clinic visit and that a repeat test be offered to initially HIV-negative women during the third trimester. Mothers were placed on lifelong highly active antiretroviral therapy (HAART) if their CD4 count was less than 350 cells/mm^3^, but if it was higher, they were placed on prophylactic antiretroviral therapy with azidothymidine (AZT) from 14 weeks of pregnancy (or at first contact with antenatal services, if later) and AZT prophylaxis was continued through labor and 1 week after delivery. HIV-exposed infants born to mothers not on HAART were prescribed nevirapine prophylaxis at birth, and this treatment was continued until 1 week after the complete cessation of breastfeeding, while for those with mothers on HAART, nevirapine prophylaxis was stopped at 6 weeks of life. Infants aged less than 18 months were tested for HIV by PCR at 6 weeks after birth or at the earliest opportunity; subsequently, an antibody test was performed at age 9 months (if the infant had previously been PCR negative) and 18 months. Infants with confirmed HIV infection at any of these test points were immediately put on HAART. All HIV-exposed infants were given prophylactic co-trimoxazole during the first 18 months of life, and those testing HIV positive at any of the testing time points continued on co-trimoxazole lifelong. HAART and co-trimoxazole were supplied at monthly visits. The infants also received their scheduled early childhood immunizations during these visits, and their immunization cards were inspected. Pairs of HIV-infected mothers and their infants (between 3 and 18 months of age) were recruited. Infants suffering from any acute infection or malnutrition at the time of recruitment were excluded from the study. Mothers contributed a single blood sample at recruitment, while the infants were followed up longitudinally every 3 months until they were 24 months of age, with an upper limit of up to 30 months being used to cover late follow-up visits. Community controls were recruited within the same locality from cohorts under active malaria surveillance, which includes an annual cross-sectional bleed (32), and from one of the sentinel dispensaries. To minimize the potential impact of malaria exposure on the B-cell compartment (33, 34), only infants who had no reported episode of malaria following weekly home visits and were negative for Plasmodium falciparum (determined by a rapid diagnostic test) during the annual cross-sectional bleed were selected. It was not possible to follow the community controls longitudinally, and therefore, infants whose ages were similar to those of the HEU infants at the three monthly follow-up time points were recruited. The prevalence of HIV infection in adults from the study area has been estimated to be 4.1% (35).

### Ethical considerations.

Informed consent was obtained from the infants' mothers, and ethical approval was granted by the National Ethics and Review Committee, Kenya Medical Research Institute, reference 2085.

### Sample collection.

At each study-related visit, a 5-ml venous blood sample was drawn and 2 ml was used for immediate analysis of hematological parameters and B-cell subsets. Peripheral blood mononuclear cells (PBMCs) and plasma were separated from the remaining 3 ml and stored in liquid nitrogen and at −80°C, respectively, until use. The single sample obtained from the mothers at their recruitment was used to determine the maternal viral load and CD4 counts at recruitment.

### Multiparametric flow cytometry.

B-cell subsets were described using the following monoclonal antibodies: fluorescein isothiocyanate (FITC)-IgM, electron-coupled dye (ECD)–CD19, and phycoerythrin-Cy5 (PC5)–CD27 (Beckman Coulter); phycoerythrin (PE)-CD21 and PE-Cy7-CD38 (eBioscience); and PE-CD10 and allophycocyanin-CD21 (BD Pharmingen). Fifty microliters of whole blood was washed and incubated with cocktails of the antibodies listed above, and the erythrocytes were lysed. At least 80,000 lymphocytes were acquired on a CyAn ADP analyzer (Beckman Coulter), and data were analyzed using FlowJo software, version 9.4.2 (TreeStar Inc., FlowJo Africa). The gating strategy used to identify different B-cell subsets is described in Fig. S1 in the supplemental material. B-cell subsets were then represented as a proportion of the total B-cell percentage in lymphocytes. Absolute B-cell subset counts were determined on the basis of the subset proportion in the total number of B cells. The total number of B cells was determined as a proportion of the absolute lymphocyte counts determined from a whole-blood cell count assay.

### ELISpot assay.

Antigen-specific IgG memory B cells (MBCs) against tetanus toxoid (TT; Statens Serum Institut), measles virus antigen (Meridian Life Science), diphtheria toxoid (DT; Alpha Diagnostics International), and pneumococcal capsular polysaccharides (PCPs) comprising a pool of 6 common serotypes within the study region (serotypes 19F, 5, 1, 23F, 14, and 6B; ATCC) (36) were quantified using a modification of a previously reported enzyme-linked immunosorbent spot (ELISpot) assay (37, 38). Briefly, 2 × 10^5^ PBMCs per well were stimulated for 5 days with 2.5 μg/ml CpG oligodeoxynucleotide-2006 (Hycult Biotech), 1:5,000 Staphylococcus aureus Cowan strain protein A (Sigma), and 83 ng/ml pokeweed mitogen (Sigma) in flat-bottomed 96-well culture plates. MultiScreen plates (Millipore) were precoated with either 5 μg/ml TT, 5 μg/ml measles virus antigen, 5 μg/ml DT, a pool of 6 PCPs each at a concentration of 10 μg/ml, 10 μg/ml polyclonal sheep anti-human IgG (Binding Site), or 1% bovine serum albumin (BSA). Cultured PBMCs were seeded onto antigen-coated plates either at 2 × 10^5^ cells/well (antigen-specific responses) or at 2 × 10^2^ or 2 × 10^3^ cells/well (total IgG responses) and incubated overnight. Alkaline phosphatase-conjugated donkey anti-human IgG antibody (Jackson ImmunoResearch Laboratories) was used as the secondary antibody. Spots were developed using 5-bromo-4-chloro-3-indolylphosphate–nitroblue tetrazolium (Bio-Rad) and counted using a CTL Immunospot analyzer (Cellular Technologies). The background was accounted for by subtracting the average number of spots in wells coated with 1% BSA from the number of spots in antigen-coated wells. An upper limit of three spots was detected at any time in the wells coated with 1% BSA.

### ELISA.

Human IgG antibodies specific to TT, DT, and a pool of 6 PCPs, described above, were quantified using a modified enzyme-linked immunosorbent assay (ELISA) protocol previously reported (39). Antibody levels were measured after the infant's 18th month of life to avoid maternally transferred antibody bias. In brief, ELISA plates were coated overnight with either TT (1 μg/ml), DT (5 μg/ml), anti-human IgG (10 ng/ml), or a pool of 6 PCPs (each at 10 μg/ml). Plasma samples were diluted at 1:1,000. Peroxidase-conjugated donkey anti-human IgG (Jackson ImmunoResearch) was used as the secondary antibody before development of the plates with *o*-phenylenediamine dihydrochloride (Sigma). The results are represented as arbitrary antibody units generated from a standard curve on the basis of the results for a control sample that was reactive to the antigen of interest. The control sample was obtained from an adult with a known vaccination history and status of reactivity to the antigen of interest.

In addition, the quantities of IgG antibodies against Haemophilus influenzae type b (Hib; Binding Site), measles virus antigen IgG ELISA (catalog number ESR102G; Serion), and respiratory syncytial virus (RSV) IgG (catalog number ESR113G; Serion) were determined by following the manufacturer's instructions.

### Maternal viral load determination.

Maternal viral loads were determined at the point of infant recruitment at the International Centre for Reproductive Health, Mombasa, Kenya, using a reverse transcription-quantitative PCR test developed by the Agence Nationale de Recherches sur le SIDA (ANRS). The assay targets a conserved long terminal repeat region and has a detection limit of 300 RNA copies/ml (40).

### Sources of data and analysis.

Stata software, version 13.1 (Stata Corporation), was used for the statistical analysis. To determine the impact of HIV exposure on infant immunological outcomes, linear regression models adjusted for clustering within a child were used. These models accounted for inherent correlations between repeated measurements done on the same subject, in addition to accounting for changes with age. To avoid maternal antibody interference, levels of antibodies against the antigens in common Expanded Programme on Immunization vaccines were determined after 18 months only. Since this aspect of the study was cross-sectional, antibody levels were compared between HEU and HUU infants using the Wilcoxon rank sum test. Mothers' clinical data, including age, body mass index (BMI; with a BMI of <18.5 being considered malnutrition), CD4 T-cell count, and HAART use, were routinely captured at the clinic. Maternal data available in the mother's records at a time point closest to that after the infant's date of birth were included in a linear regression model to determine the impact of maternal health close to the time of the infant's birth on the infant's developing immune system. Only data collected within the first 4 months after the infant's birth were considered. In addition, the mother's CD4 count, BMI, and viral load were collected during the infant's recruitment and were included in the linear regression model to determine the impact of the mother's health during this period on the infant's developing immunity.

## RESULTS

### Baseline characteristics of study population.

Between November 2011 and December 2012, infants born to HIV-infected mothers were enrolled at the CCRC, Kilifi County Hospital. Of the 92 infants recruited, 5 (5.4%) tested positive for HIV. Two tested positive at 6 weeks of age, while the remaining 3 were found to be positive at the first clinic visit, which was made more than 2 months after birth (see Fig. S2 in the supplemental material). The HIV-infected infants were excluded from the study, and the subsequent analysis concentrated on HIV-exposed uninfected infants. Forty-three out of 87 of the HEU infants (49.4%) were boys. Ninety-eight community controls under 30 months of age were recruited for comparison. Nine of them were sampled twice, having participated in two annual cross-sectional bleeds. Data for the HEU infants' mothers recorded closest to the time after the infants' date of birth and collected during recruitment were available (Table 1). The majority of the mothers had a BMI of >18.5 (and, hence, were considered well nourished) both at the time closest to the time after the infant's date of birth and at recruitment. They also had CD4 counts above 400 cells/mm^3^ at both time points. We were able to obtain viral load data for 78 of the mothers. Of these, 38/78 (48.7%) had viral loads less than 300 copies per ml and 25/78 (32.1%) had viral loads above 5,000 copies per ml. Mothers who had been on HAART for more than 2 years prior to the infant's birth had a lower median viral load at recruitment than those who had not been on HAART (Table 1).

**TABLE 1:** Data for mothers of HIV-exposed uninfected infants taken at the time closest to the time after the infant's date of birth and during the infant's recruitment^*a*^

Characteristic	Result for mothers:	
	Close to time of infant's birth	During infant's recruitment
Median (IQR) age (yr) (*n* = 87)	29.2 (25.4–34.2)	30.1 (26.1–35.1)
Median (IQR) BMI^*b*^	21.5 (19.5–23.3)	21.2 (19.5–23.0)
Median (IQR) CD4 count (no. of cells/mm^3^)^*c*^	410 (292–640)	457 (322–603)
% of mothers with the following viral load (no. of copies/ml)^*d*^:		
<300	ND	48.7 (38/78)
>300–<1,000	ND	7.7 (6/78)
>1,000–<5,000	ND	11.5 (9/78)
>5,000	ND	32.1 (25/78)
Median (IQR) viral load (no. of copies/ml) categorized by HAART use close to time of infant's birth		
Not on HAART (*n* = 37)	ND	2,783 (54–20,685)
HAART for 0–24 mo (*n* = 13)	ND	29 (0–2,648)
HAART for >24 mo (*n* = 20)	ND	10 (0–402)
Missing HAART category (*n* = 8)^*e*^	ND	2,428 (18–25,894)

a HAART, highly active antiretroviral therapy; IQR, interquartile range; ND, not determined.

b Data are for 72 mothers at the time close to the time of the infant's birth and 74 mothers during the infant's recruitment.

c Data are for 53 mothers at the time close to the time of the infant's birth and 75 mothers during the infant's recruitment.

d Viral load data were available for 78 of the mothers. Data in parentheses represent the number of mothers with the indicated viral load/total number of mothers tested.

e Mothers for whom data on the duration on HAART close to the time of the infant's birth were missing.

### HIV exposure is associated with a reduced proportion of unswitched MBCs.

The first 70 HEU infants to be recruited were included in the B-cell phenotypic analysis. These infants were not different from the remaining 17 who were recruited later and not included in the B-cell phenotypic analysis. We analyzed the B-cell subsets in 140 peripheral blood samples from these 70 infants (34 HEU infants contributed one sample, and 36 contributed multiple samples) and 98 HUU infants (9 infants were bled at two annual cross-sectional bleeds and therefore contributed two samples each) (see Table S1 in the supplemental material) to determine whether HIV exposure is associated with an altered B-cell subset distribution. In the univariate regression analysis, HIV exposure was associated with a significant reduction in the total B-cell proportion, largely due to changes in the memory B cell (MBC) compartment (Table 2). A reduction in resting memory B cells (CD19^+^ CD10^−^ CD27^+^ CD21^+^) was associated with HIV exposure. This association was not observed when the whole memory B cell population (CD19^+^ CD10^−^ CD27^+^) was considered. It was largely due to changes in the unswitched MBC subset (CD19^+^ CD10^−^ CD27^+^ IgM positive [IgM^+^]), while the proportions of switched MBCs (CD19^+^ CD10^−^ CD27^+^ IgM negative [IgM^−^]) were similar in HEU and HUU infants. The association of HIV exposure with a lower proportion of unswitched MBCs was maintained even after adjusting for multiple testing using the Bonferroni correction and correcting for age. Similarly, when absolute B-cell subset counts were considered, HIV exposure was significantly associated with a reduction of unswitched absolute MBC counts (Table 2).

**TABLE 2:** Association of HIV exposure and changes in infants' B-cell subset percentages and absolute counts during first 2 years of life^*a*^

B-cell subset	Effect parameter	B-cell subset %				B-cell subset absolute no.			
		Univariate linear regression		Multivariate linear regression		Univariate linear regression		Multivariate linear regression	
		β coefficient (SE)	*P* value	β coefficient (SE)	*P* value	β coefficient (SE)	*P* value	β coefficient (SE)	*P* value
B cells	HIV exposure	**−0.139 (0.054)**	**0.012**	**−0.138 (0.055)**	**0.013**	−0.063 (0.089)	0.482		
	Age	−0.004 (0.004)	0.35	−0.004 (0.004)	0.378	−0.0126 (0.0067)	0.061		
Naive B-cells	HIV exposure	−0.005 (0.017)	0.760			−0.019 (0.098)	0.850		
	Age	**−0.008 (0.001)**	**<0.001**			**−0.020 (0.007)**	**0.004**		
Total MBCs	HIV exposure	−0.152 (0.110)	0.168			−0.054 (0.122)	0.657		
	Age	**0.800 (0.062)**	**<0.001**			**−1.430 (0.131)**	**0.000**		
Resting MBCs	HIV exposure	**−0.211 (0.086)**	**0.015**	**−0.259 (0.065)**	**<0.001**	−0.191 (0.124)	0.124		
	Age	**1.531 (0.093)**	**<0.001**	**0.812 (0.045)**	**<0.001**	**−1.351 (0.161)**	**<0.001**		
Unswitched MBCs	HIV exposure	**−0.337 (0.095)**	**<0.001**	**−0.359 (0.081)**	**<0.001**	**−0.287 (0.138)**	**0.039**	**−0.364 (0.128)**	**0.005**
	Age	**0.620 (0.061)**	**<0.001**	**0.629 (0.057)**	**<0.001**	**−0.466 (0.067)**	**<0.001**	**−0.493 (0.061)**	**<0.001**
Switched MBCs	HIV exposure	0.131 (0.089)	0.140			0.201 (0.126)	0.111		
	Age	**0.752 (0.046)**	**<0.001**			**−1.34 (0.173)**	**<0.001**		
Atypical MBCs	HIV exposure	0.133 (0.081)	0.100			0.130 (0.116)	0.266		
	Age	0.009 (0.005)	0.103			−0.038 (0.019)	0.052		
Activated B cells	HIV exposure	−0.022 (0.077)	0.780			−0.050 (0.106)	0.634		
	Age	**−0.954 (0.114)**	**<0.001**			**−0.374 (0.064)**	**<0.001**		
Plasmablasts	HIV exposure	0.040 (0.144)	0.782			0.0370 (0.164)	0.823		
	Age	**0.037 (0.009)**	**<0.000**			**−0.335 (0.103)**	**0.001**		

a Linear regression models were used to describe the estimated change in an infant's B-cell subset percentages and counts (β coefficients with standard errors). The impact of HIV exposure on B cells was determined at a significance level of a *P* value of <0.05. Subset analysis on naive B cells, total memory B cells, and plasmablasts was performed after adjusting for multiple testing using the Bonferroni correction at a significance level of a *P* value of <0.02, and further subgroup analysis within the memory B cells (resting, atypical, activated, switched, and unswitched memory B cells) was performed at a significance level of a *P* value of <0.005. Statistically significant values are shown in boldface type. The linear regression models were adjusted for clustering within an infant and, hence, accounted for inherent correlations between repeated measurements done on the same subject. MBC, memory B cell.

**FIG 1:**
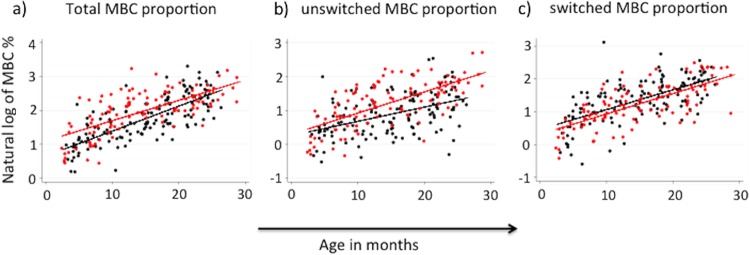
Distribution of memory B cells (CD19^+^ CD10^−^ CD27^+^) (a), unswitched memory B cells (CD19^+^ CD10^−^ CD27^+^ IgM^+^) (b), and switched memory B cells (CD19^+^ CD10^−^ CD27^+^ IgM^−^) (c) in HUU infants (red dots) and HEU infants (black dots). Straight lines show the best-fit prediction of the increment in subset proportions over the 2 years of life. The percentage of memory B cell subsets is presented on the *y* axis as the natural log of the MBC percentages.

Although memory B cells gradually accumulated with age in HEU infants, as observed from individual infants' kinetics for those who had data from more than one time point (see Fig. S4 in the supplemental material), when all infants were considered (Fig. 1), HIV exposure resulted in a slower development of the memory B-cell compartment (Fig. 1). In line with the overall reduction in unswitched memory B cells, this was true for the unswitched MBC subset, while the gradual accumulation in the switched MBC compartment observed in HEU infants remained comparable to that observed in HUU infants during these first 2 years of life.

We further determined whether a mother's data recorded closest to the time after her infant's date of birth and/or at recruitment had an impact on the B-cell subset distribution observed in the HEU infants. There was no association between the majority of the maternal parameters and the infant's B-cell subset distribution, apart from the total B-cell percentage, which directly correlated with maternal CD4 counts but inversely with the maternal BMI closest to the time after the infant's date of birth and the BMI at infant recruitment. Interestingly, the maternal viral load, although measured only at the infant's recruitment, was significantly associated with higher proportions of infant plasmablasts and MBC subsets but a lower proportion of naive B cells (see Table S2 in the supplemental material), suggesting a higher level of immune activation in infants of mothers with ongoing viral replication.

### Recall responses to selected vaccine antigens are comparable between HEU and HUU infants.

We measured recall responses to representative antigens (measles virus antigen, PCPs, DT, TT, and total IgG) by ELISpot assay in 64 HEU infants (22 infants contributed a single sample, and 18 infants contributed more than one sample) and 29 HUU infants (the samples used are described in Table S1 in the supplemental material). The samples selected for these analyses were limited by the availability of adequate cell numbers in samples from HUU infants. In linear univariate analysis, HIV exposure was associated with a reduction in total IgG^+^ MBC recall responses (Table 3). This association was maintained even after adjusting for age. However, HIV exposure did not perturb the generation of antigen-specific IgG MBC recall responses against measles virus antigen, PCPs, DT, and TT, which were similar in HEU and HUU infants. The mothers' data recorded closest to the time after the infants' dates of birth and at recruitment were not associated with an infant's ability to generate recall responses to previously encountered antigens (see Table S2 in the supplemental material).

**TABLE 3:** Impact of HIV exposure on infants' memory B-cell recall responses during the first 2 years of life to antigens that they had previously been vaccinated against^*a*^

Antigen	Effect parameter	Univariate analysis		Multivariate analysis	
		β coefficient (SE)	*P* value	β coefficient (SE)	*P* value
PCPs	HIV exposure	0.713 (0.685)	0.302		
	Age	0.0813 (0.068)	0.235		
Measles virus	HIV exposure	0.309 (1.033)	0.766		
	Age	0.024 (0.197)	0.904		
TT	HIV exposure	−0.195 (1.049)	0.853		
	Age	0.059 (0.074)	0.431		
DT	HIV exposure	0.708 (0.727)	0.334		
	Age	−0.022 (0.056)	0.700		
Total IgG	HIV exposure	**−44.44 (20.88)**	**0.037**	**−48.145 (19.14)**	**0.014**
	Age	**7.268 (1.212)**	**0.000**	**7.418 (1.266)**	**0.000**

a Linear regression models were used to describe the estimated change in an infant's B-cell recall responses to antigens that they had been previously vaccinated against (β coefficients with standard errors). *P* values less than 0.05 were considered significant and are indicated in bold. B-cell recall responses whose changes were significantly associated with HIV exposure were included in a multivariate regression model accounting for age. The linear regression models adjusted for clustering within an infant and, hence, accounted for inherent correlations between repeated measurements done on the same subject.

### Levels of IgG antibodies to selected vaccine antigens at 18 months of life.

**FIG 2:**
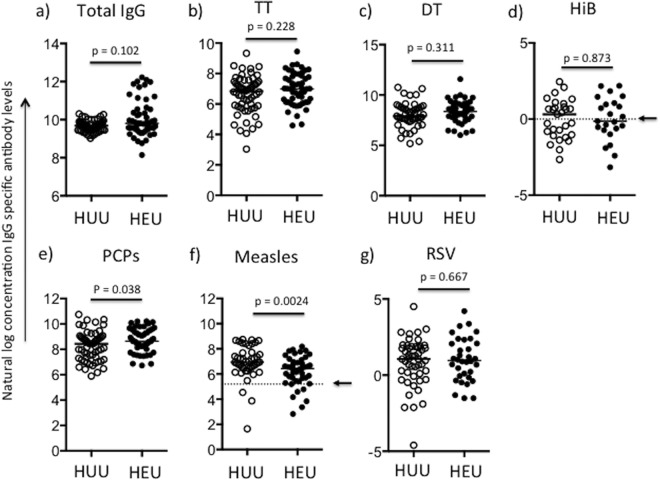
Levels of IgG antibodies to total IgG (a) and selected vaccine antigens, TT protein (b), diphtheria toxin (c), Hib (d), PCPs (e), measles virus antigen (f), and RSV (g), at 18 months of life. Antibody concentrations were compared between HUU infants (open circles) and HEU infants (closed circles). The Wilcoxon rank sum test was used, and medians are presented. *P* values of <0.05 were considered significant. Arrows in panels d and f, cutoff for protective antibody concentration. Since antibody concentrations were not normally distributed, natural log-transformed values of arbitrary antibody concentrations (TT, diphtheria toxin, PCPs, total IgG) and absolute concentrations (measles virus antigen [in mIU/ml], Hib [in mg/liter], and RSV [in U/ml]) are presented.

Next, we assessed whether the plasma concentrations of antibodies to vaccine antigens and the agents of common childhood infections were comparable between HEU and HUU infants. The maintenance of levels of antibodies to selected vaccine antigens was determined after 18 months of life in 55 and 48 plasma samples from HEU and HUU infants, respectively. The samples used are described in Table S1 in the supplemental material. A wide variation in total IgG antibody levels was observed in the HEU group compared to the variation observed in the HUU group (Fig. 2a). Out of four antigens (TT, DT, Hib, and PCPs) against which vaccination is given in the first 16 weeks of life, anti-PCP IgG levels were significantly higher in the HEU infants than the HUU infants (Mann-Whitney U test *P* value = 0.038) (Fig. 2b to e). The levels of antibodies against measles virus antigen, against which infants are vaccinated in the 9th month of life, were lower in the HEU infants than the HUU infants (Mann-Whitney U test *P* value = 0.0024) (Fig. 2f). Nevertheless, the majority of infants in both groups attained the conventionally accepted protective antibody level of 200 mIU/ml (41). No differences in the levels of IgG antibodies against RSV, to which infants are naturally exposed, were observed (Fig. 2g). Of concern, approximately 50% of both HEU and HUU infants' anti-Hib antibody levels were below a threshold required for long-term protection by 18 months of life (42) (Fig. 2d). For the two antigens against which antibody levels were observed to be significantly different between HEU and HUU infants, measles virus antigen and PCPs, a subanalysis of antibody levels at 18 months of age and after 18 months of age (21 and 24 months) was done. Antibodies against PCPs showed an age-related increase, with the levels being significantly higher in HEU infants at a time when they were no longer receiving co-trimoxazole prophylaxis (see Fig. S3 in the supplemental material). Comparison of antigen-specific antibody levels between HEU infants whose mothers had been on HAART or not at the time of the infant's birth (that is, mothers who had been receiving antivirals besides azidothymidine [AZT] prophylaxis, given per the Kenyan guidelines at the time of the study [30]) showed no significant differences, but total IgG antibody levels were higher in infants whose mothers had received HAART (Mann-Whitney U test *P* value = 0.034; data not shown). When maternal data recorded closest to the time after the infant's birth and at recruitment were considered, there was no association with the infant's ability to maintain antibodies against antigens they had been vaccinated against (see Table S2 in the supplemental material).

### The total MBC percentage correlates with antigen-specific MBC numbers and antibody levels.

**FIG 3:**
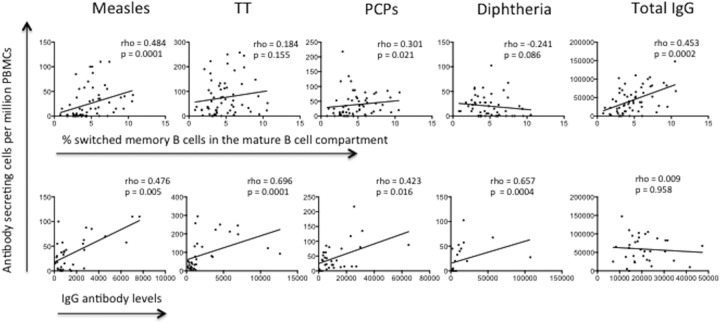
Correlation of circulating antigen-specific memory B cells with the percentage of switched memory B cells (first row) or levels of IgG antibodies (second row) against measles virus antigen, TT protein, PCPs, diphtheria toxin, and total IgG. Spearman correlation coefficients were determined, and the Spearman rho values are presented. *P* values of <0.05 were considered significant.

Data on the distribution of total B-cell subsets, the antigen-specific MBC recall response, and levels of antibodies to vaccine antigens were available for 64 HEU infants and 29 HUU infants. We correlated these data to determine if infants who had a higher percentage of switched MBCs were better at generating recall responses or had higher antibody levels in their circulation. Infants' recall responses to measles virus antigen, PCPs, and total IgG-secreting B cells directly correlated with the percentage of MBCs in their peripheral blood circulation (Fig. 3). In addition, recall responses to measles virus antigen, TT, PCPs, and DT directly correlated with the levels of antibodies against the same antigens. For the community controls for which phenotypic, recall response, and antibody data were available, recall responses to TT and PCPs correlated with antibody levels (Spearman correlation coefficient rho values were 0.600 and 0.665, respectively, and *P* values were <0.039 and 0.036, respectively; data not shown).

## DISCUSSION

We undertook a comprehensive analysis of the impact of HIV exposure on the developing B-cell compartment in HIV-exposed uninfected infants. Infants born to HIV-infected mothers, even when the infants are not infected, may be exposed to HIV antigens and HAART *in utero* and during breastfeeding (43, 44).

Only a few studies have addressed the impact of HIV exposure on the B-cell compartment (16, 27–29). A recent report on Malawian HEU infants showed similar B-cell subsets in HEU infants and HUU infants during the first 18 months of life. In agreement with that finding, the majority of the B-cell subsets in our study were similar in HEU infants and HUU infants. However, we observed an association between HIV exposure and a reduced proportion of unswitched MBCs. Although the existence of unswitched (IgM^+^) memory B cells was previously debated (45, 46), there is increasing evidence of their existence and their ability to undergo secondary germinal center reactions and receive T-cell help (47). A recent study showed that unswitched memory B cells play a special role in early inflammation through their interaction with immunomodulatory neutrophils (48). Additionally, it has been suggested that unswitched memory B cells preferentially reenter germinal centers upon antigen reactivation and, hence, play an active role in sustaining memory, while switched memory B cells show a propensity to differentiate directly into plasmocytes (48–50).

In our study setup, it is possible that MBC responses to natural antigens developed more slowly in these HEU infants due to the daily co-trimoxazole prophylaxis, routinely given, which may reduce the infant's exposure to a broad spectrum of pathogens, as it is intended to do (30). However, some studies have reported a lack of reduction of pneumococcal nasopharyngeal carriage in HIV-infected children despite co-trimoxazole prophylaxis, suggesting that the direct effect on exposure to some bacterial infections could be limited even in HEU infants (51). Co-trimoxazole may have led to some immunomodulatory mechanisms that may have resulted in reduced lymphocyte proliferation (52). It is also possible that HEU infants intrinsically develop a smaller unswitched B-cell compartment.

The lower proportion of IgM memory B cells in HEU infants may have clinical consequences, compromising their first-line humoral responses and, hence, predisposing them to infections (53). While switched memory B cells dominate the secondary response due to their capacity to be activated in the presence of neutralizing serum immunoglobulin, it appears that once the levels of neutralizing antibodies drop, memory is sustained by IgM reserves (50). A lower proportion of IgM MBCs in HEU infants may therefore interfere with their ability to sustain long-term memory should levels of protective antibodies fall below a threshold. Although determination of the clinical consequences of immunological changes in the HEU infants was beyond the scope of the current study, our observation warrants further investigation to determine if the smaller amounts of unswitched MBCs in HEU infants, even in the presence of switched MBCs in amounts similar to those in HUU infants, possess any clinical consequences.

It is encouraging that similar proportions of switched MBCs were observed in HEU and HUU infants, implying that HEU infants are capable of mounting robust responses to vaccine antigens. Lower levels of interference from maternal antibodies may also have contributed to the robust responses, as has been previously suggested (21). In support of the HEU infants' ability to mount robust vaccine responses, recall responses to previously encountered vaccine antigens were similar in HEU and HUU infants.

For a majority of the vaccine antigens against which the levels of antibodies were determined, HEU infants were able to maintain antibody levels similar to those observed in HUU infants, as previously reported in other settings (21, 22). However, we observed significantly lower levels of antibodies against measles virus antigen in the HEU infants, but of importance, the majority of these infants attained the recommended protective level (41). HEU infants had higher antipneumococcal antibody levels than HUU infants after age 18 months. From previous reports, HIV-infected women with opportunistic infections might be more likely to transmit these infections to their infants (54, 55). It is therefore likely that environmental exposure from an ailing mother may have led to increased exposure, leading to the observed higher antipneumococcal antibody levels once co-trimoxazole prophylaxis stopped at 18 months of age.

Of concern, in our study population, a large proportion of both the HEU and HUU infants showed levels of antibodies against Haemophilus influenzae type b below a threshold deemed protective, an observation made previously (23). It is possible that MBCs rather than serological memory sustain protection (56), although sustained antibody levels at age 24 months have been reported (22). This implies that antibodies may also play a role and a booster dose after early infancy may be beneficial to both HEU and HUU infants. In our study, we concentrated on antibody levels, and it may be important for future studies to incorporate antibody functional assays, which would comprehensively ascertain if the HIV-exposed infants are compromised.

The results of analyses of the correlation between the serological and recall response data, although they are from a sample with a modest size and therefore should be interpreted with caution, suggest that infants who made good long-lasting antibody responses also made better recall responses. The degree of overlap between the memory B-cell compartment and long-lived antibody-secreting cells, plasma cells, is difficult to determine (57), and both compartments may play important, albeit different, roles. Given that the infants were in the first 2 years of life, it is likely that natural exposure was minimal, and therefore, it is more likely for antibody and memory function responses to correlate (58). Increased antigenic exposure with age may lead to a loss of this correlation.

The delayed accumulation of memory B cells in HEU infants observed in this study warrants further investigation. A recent report from Malawi showed no differences in the proportions of memory B cells between HEU infants and HUU infants. Although the procedures of clinical care for HEU infants are similar in these two settings, levels of exposure to the malaria parasite, which is also known to perturb the B-cell compartment (33) in a manner similar to that in which HIV perturbs that compartment (18, 19), may have been different. Perturbation of the generation and maintenance of B-cell memory in malaria parasite infection (59) and distortion of the B-cell compartment with the appearance of additional subsets not commonly found in healthy individuals (33, 60) have been reported. We selected HUU infants with no previously reported clinical episode of malaria on the basis of active weekly surveillance. This ensured that they were more comparable to the HEU infants who received daily co-trimoxazole prophylaxis, which, although not the primary aim, protects them from malaria (61, 62). This may have increased our chances of identifying subtle immunological differences associated with HIV exposure, which may be missed in settings where perturbations in HUU infants may have already been caused by malaria exposure.

In the future, studies on the potential role of HIV exposure on the infant's developing immune system will have to be carefully designed to take into account various environmental exposures, such as malaria endemicity and PMTCT programs, which, for example, call for the daily use of co-trimoxazole prophylaxis, to comprehensively conclude if HIV exposure perturbs the infant's developing immune system.

Our findings describe B-cell memory vaccine responses and complement the current body of data on serological responses that have shown sustained antibody levels in exposed infants up to 2 years of age (22) and recent findings on B-cell phenotypes in HEU infants (29). In our study, though, HIV exposure had subtle effects on the development of the B-cell compartment and was significantly associated with a reduction in the unswitched memory B-cell proportions. Our findings also imply that maternal health may impact the infants' responses, particularly to pneumococcal antigens. Placing mothers on lifelong HAART earlier may contribute to reduced vulnerability to infections in general and benefit both maternal and infant health. Importantly, our study shows that exposed infants mount robust B-cell responses to vaccines and pathogens and that it is therefore likely that these infants would be able to respond to a future HIV vaccine to prevent infection in this at-risk population.

## Supplementary Material

Supplemental material
