# Supplementary material for: HIV-Exposed Uninfected Infants Show Robust Memory B-Cell Responses in Spite of a Delayed Accumulation of Memory B Cells: an Observational Study in the First 2 Years of Life
Source: Clin Vaccine Immunol. 2016 Jul 5;23(7):576–85. doi: 10.1128/CVI.00149-16 (PMC4933775; doi:10.1128/CVI.00149-16)
Supplement: Supplemental material [file supp_23_7_576__index.html]

Supplemental material 

# HIV-Exposed Uninfected Infants Show Robust Memory B-Cell Responses in Spite of a Delayed Accumulation of Memory B Cells: an Observational Study in the First 2 Years of Life

## Supplemental material

- Supplemental file 1 -

  Fig. S1. Gating strategy for the distribution of B-cell subsets by multiparametric flow cytometry based on various combinations of surface markers. Fig. S2. Infant recruitment and numbers of infants included in the B-cell phenotypic analysis, recall/memory B-cell analysis, and antibody level analysis. Fig. S3. Comparison of antipneumococcal capsular polysaccharide and antimeasles antibody levels taken at and after 18 months. Fig. S4. Memory B-cell subset kinetics in individual infants during the first 2 years of life. Table S1. Number of samples used to determine B-cell phenotypes, B-cell recall/memory responses, and serological responses. Table S2. Association of maternal data taken within the first 4 months after the infant’s birth and at recruitment with the infant’s immunological outcomes.

  PDF, 1.1M
