# Supplementary material for: HIV-Exposed Uninfected Infants Show Robust Memory B-Cell Responses in Spite of a Delayed Accumulation of Memory B Cells: an Observational Study in the First 2 Years of Life
Source: Clin Vaccine Immunol. 2016 Jul 5;23(7):576–85. doi: 10.1128/CVI.00149-16 (PMC4933775; doi:10.1128/CVI.00149-16)
Supplement: Supplemental material [file CVI.00149-16_zcd999095367so1.pdf]

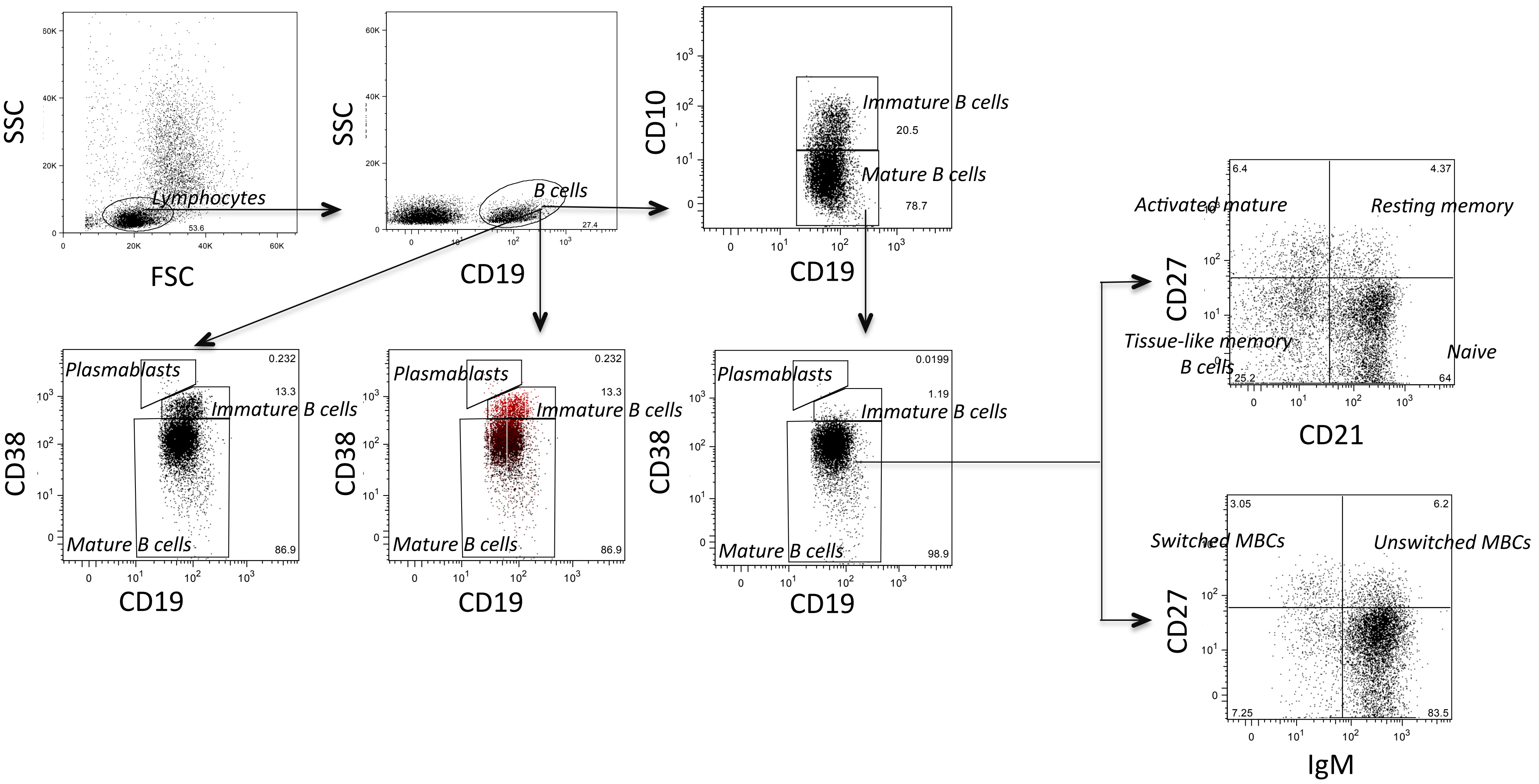
SSC
FSC
Lymphocytes
53.6
SSC
CD19
B cells
27.4
CD10
CD19
Immature B cells
20.5
Mature B cells
78.7
CD27
CD21
Activated mature
Resting memory
Tissue-like memory
B cells
Naive
6.4
4.37
25.2
64
CD38
CD19
Plasmablasts
Immature B cells
Mature B cells
0.232
13.3
86.9
CD38
CD19
Plasmablasts
Immature B cells
Mature B cells
0.232
13.3
86.9
CD38
CD19
Plasmablasts
Immature B cells
Mature B cells
0.0199
1.19
98.9
CD27
IgM
Switched MBCs
Unswitched MBCs
3.05
6.2
7.25
83.5

Fig S1

Supplementary Figure 1:

Gating strategy for the distribution of B-cell subsets by multiparametric flow cytometry based on the combination of surface markers as indicated. B cells; CD19^{+}, plasmablasts; CD19^{+}CD38^{hi}CD10^{-}, mature B cells; CD19^{+}CD38^{lo}CD10^{-}, naïve B cells; CD19^{+}CD10^{-}CD27^{-}CD21^{+}, resting MBCs; CD19^{+}CD10^{-}CD27^{+}CD21^{+}, activated B cells; CD19^{+}CD10^{-}CD27^{+}CD21^{-}, tissue-like MBCs; CD19^{+}CD10^{-}CD27^{-}CD21^{-}, switched MBCs; CD19^{+}CD10^{-}CD27^{+}IgM^{-}, unswitched MBCs; CD19^{+}CD10^{-}CD27^{+}IgM^{+}. *Gating on the CD10^{-}CD19^{+} population excludes the immature B cells (CD38^{+}CD19^{+}CD10^{+}) as confirmed by the polychromatic plot CD10^{+} cells (red dots). MBCs: memory B cells.

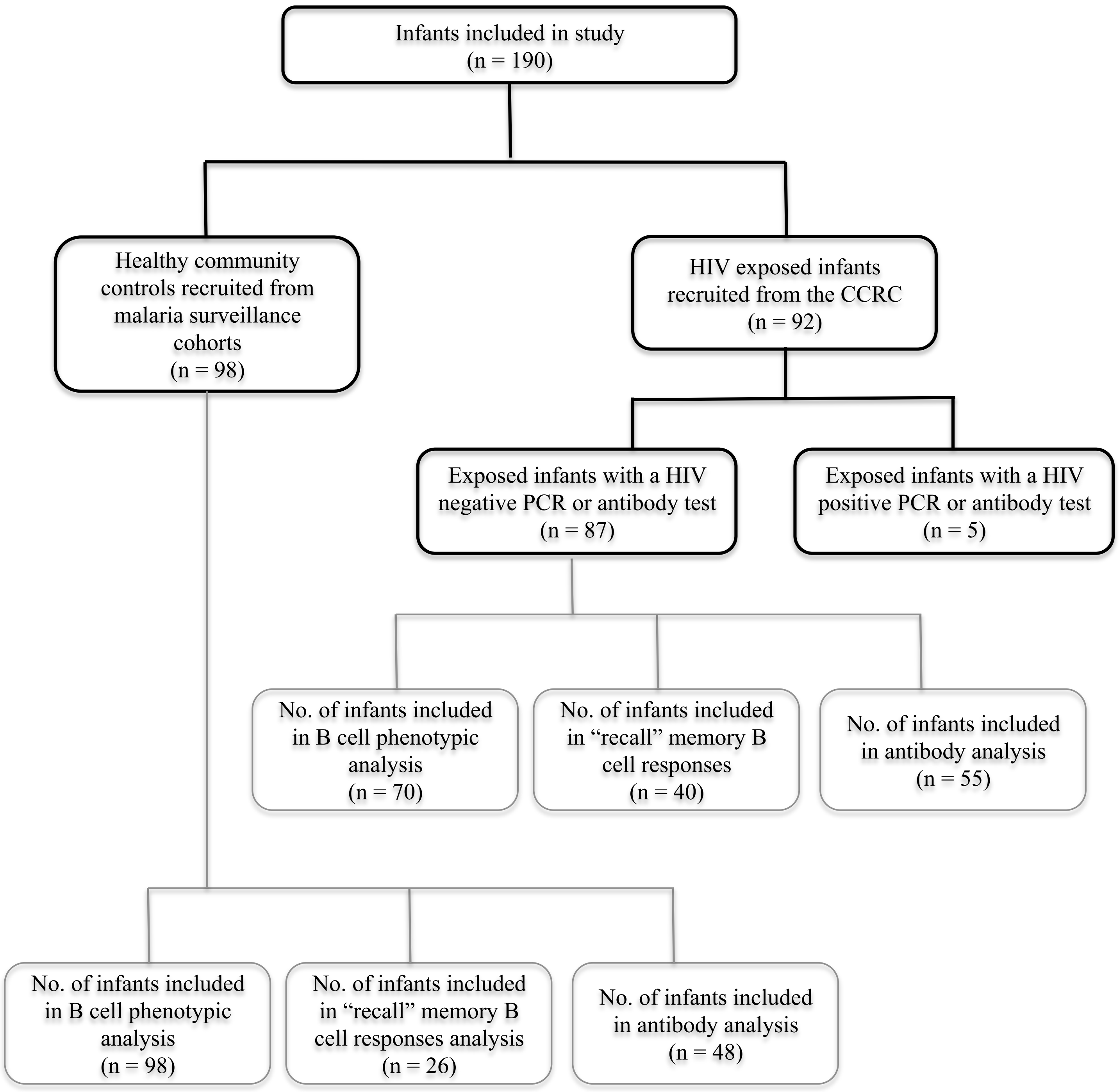
Infants included in study
(n = 190)
Healthy community controls recruited from malaria surveillance cohorts
(n = 98)
HIV exposed infants recruited from the CCRC
(n = 92)
Exposed infants with a HIV negative PCR or antibody test
(n = 87)
Exposed infants with a HIV positive PCR or antibody test
(n = 5)
No. of infants included in B cell phenotypic analysis
(n = 70)
No. of infants included in "recall" memory B cell responses
(n = 40)
No. of infants included in antibody analysis
(n = 55)
No. of infants included in B cell phenotypic analysis
(n = 98)
No. of infants included in "recall" memory B cell responses analysis
(n = 26)
No. of infants included in antibody analysis
(n = 48)

Fig S2

Supplementary Figure 2

Infant recruitment and numbers of infants included in the B cell phenotypic analysis, recall/memory B cell analysis and antibody level analysis.

A total of 190 infants were included in the study, healthy community controls (n = 98) and HIV exposed infants (n = 92). Five of the HIV exposed infants were HIV positive and excluded from subsequent assays. Of the 87 HEUs who were negative for HIV, 70 were included in B cell phenotypic assays, a subset of these infants, 40 and 55 were also included in the recall/memory B cell assay and antibody concentration assays respectively. Amongst the healthy community controls 98 infants were included in the B cell phenotypic assays while a subset of these infants, 26 and 48, were included in recall/memory B cell and antibody assays respectively. Overall 17 HIV exposed uninfected infants were not included in the immunological assays (phenotypic, recall/memory and antibody analysis), due to delayed recruitment, but their HIV test results were used in determining the proportion of HIV exposed infants who tested HIV positive by PCR or antibody test [5 out of 92 (5.4%)] in our study.

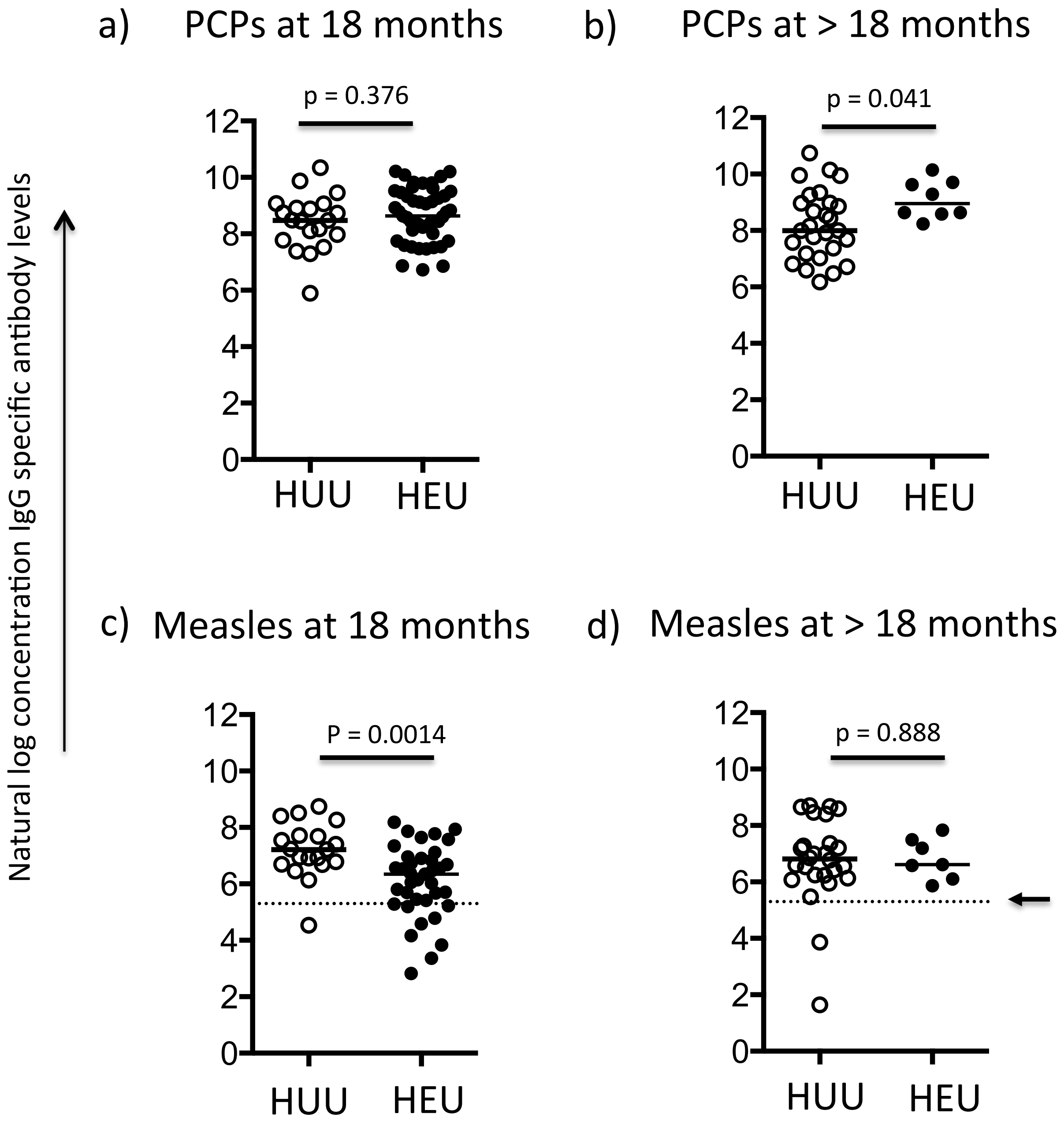
a) PCPs at 18 months
b) PCPs at > 18 months
c) Measles at 18 months
d) Measles at > 18 months
p = 0.376
p = 0.041
P = 0.0014
p = 0.888
Natural log concentration IgG specific antibody levels
0
2
4
6
8
10
12
HUU
HEU

Fig S3

Supplementary Figure 3

A comparison of anti-pneumococcal capsular polysaccharides (PCPs) and anti-measles antibody levels taken at the 18-month age-category and after 18 months (age categories 21 and 24 months).

Antibody levels against a) PCPs taken at 18 months age category, b) against PCPs taken after 18 months (age-categories 21 and 24 months) c) against measles taken at 18 months and d) against measles taken after 18 months (age-categories 21 and 24 months). Antibody concentrations were compared between HIV unexposed uninfected infants (HUU), open circles and HIV exposed uninfected infants (HEU), closed circles. Wilcoxon rank-sum test was used and medians presented. P-values < 0.05 were considered significant. Arrow and dotted lines indicate cut-off for protective antibody concentration. Since antibody concentrations were not normally distributed, log transformed (natural-log) values of arbitrary antibody concentrations (PCPs) and absolute concentrations (measles (mlU/ml) have been presented.

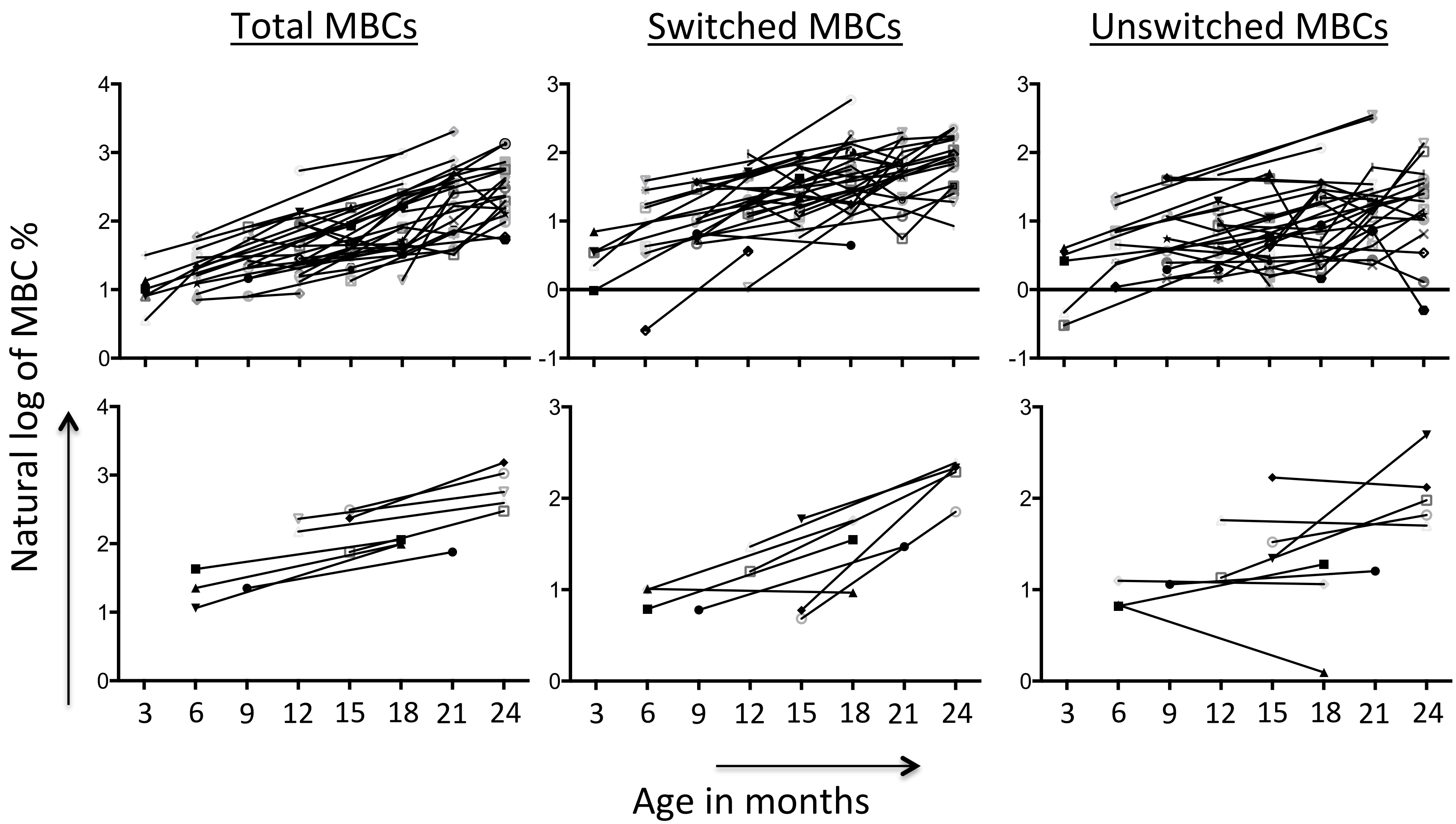
Total MBCs
Switched MBCs
Unswitched MBCs
HEUs
HUUs
Natural log of MBC %
Age in months
3 6 9 12 15 18 21 24

Fig S4

Supplementary Figure 4

Memory B-cell subset kinetics in individual infants during the first two years of life.

Individual infant's kinetics of memory B cell subset; total memory B cells ($CD19^{+}CD10^{-}CD27^{+}$), switched memory B cells ($CD19^{+}CD10^{-}CD27^{+}$ IgM^{-}) and unswitched memory B cells ($CD19^{+}CD10^{-}CD27^{+}IgM^{+}$) changes with age. In the first row; HIV exposed uninfected infants (HEU) and the second row HIV unexposed uninfected infants (HUU). Each line joins individual infant's MBC subset proportion over time. Y-axis; percentage of memory B cell subsets have been presented as the natural-log of the MBC percentages.

Supplementary Table 1: Number of samples used to determine B-cell phenotypes, B-cell recall/memory responses and serological responses.

		B-cell phenotypic description		B-cell recall responses		Antigen specific antibody levels	
		HEUs (n = 70)	HUUs (n = 98) [d]	HEUs (n = 40)	HUUs (n = 26)	HEUs (n = 55)	HUUs (n = 48)
Age categories in months	3	13	10	N/D	N/D	N/D	N/D
	6	16	14	6	3	N/D	N/D
	9	16	16	9	2	N/D	N/D
	12	14	15	9	4	N/D	N/D
	15	18	10	7	7	N/D	N/D
	18	23	13	9	7	47	20
	21	19	12	10	3	5	22
	24	21	17	14	3	3	6
	Total[c]	140	107[d]	64	29	55	48

n = number of infants contributing the samples that were analysed

[c] = total number of samples analysed from n infants

[d] = 9 community controls were sampled twice, 12 months apart during the annual cross-sectional bleed.

N/D = not determined

HEU; HIV exposed uninfected infants born of infected mothers, HUU; HIV unexposed uninfected infants born of uninfected mother

Supplementary Table 2: The association of maternal data taken within the first four months after the infant's birth and at recruitment, with infant's immunological outcomes.

	**Maternal characteristics after infant's birth**						**Maternal characteristics at infant's recruitment**					
	**CD4 count**		**BMI**		**HAART use**		**CD4**		**BMI**		**Viraemia**	
	**Beta Coef (Std Err)**	**P value**	**Beta Coef (Std Err)**	**P value**	**Beta Coef (Std Err)**	**P value**	**Beta Coef (Std Err)**	**P value**	**Beta Coef (Std Err)**	**P value**	**Beta Coef (Std Err)**	**P value**
**Infants' B cell subsets**												
B cells	**0.0006<br>(0.0003)**	**0.039**	**-0.022<br>(0.011)**	**0.058**	-0.074<br>(0.055)	0.186	5.68e-06<br>(0.0003)	0.981	**-0.24<br>(0.012)**	**0.046**	1.19e-07<br>(6.07e-08)	0.056
Naïve B cells	0.0001<br>(0.0002)	0.515	-0.004<br>(0.004)	0.307	-0.024<br>(0.025)	0.333	0.000022<br>(0.00008)	0.775	-0.0039<br>(0.0051)	0.444	**-5.65e-08<br>(2.25e-08)**	**0.015**
Resting MBCs	0.0003<br>(0.0004)	0.493	0.007<br>(0.021)	0.749	0.034<br>(0.069)	0.623	0.000037<br>(0.00033)	0.911	0.00015<br>(0.023)	0.995	**3.05e-07<br>(5.57e-08)**	**0.000**
Unswitched MBCs	0.0005<br>(0.0004)	0.204	0.013<br>(0.023)	0.615	0.007<br>(0.093	0.932	-0.00004<br>(0.0003)	0.903	0.016<br>(0.024)	0.513	**2.47e-07<br>(9.39e-08)**	**0.011**
Switched MBCs	-0.0004<br>(0.0004)	0.365	0.005<br>(0.018)	0.766	0.0755<br>(0.076)	0.322	-0.000127<br>(0.00026)	0.629	-0.012<br>(0.019)	0.522	**1.39e-07<br>(6.37e-08)**	**0.034**
Atypical MBCs	-0.0003<br>(0.0004)	0.347	0.017<br>(0.020)	0.400	0.0386<br>(0.085)	0.650	-0.00012<br>(0.0003)	0.701	0.0006<br>(0.021)	0.772	**2.72e-07<br>(1.03e-07)**	**0.011**
Activated B cells	0.0004<br>(0.0003)	0.237	0.004<br>(0.018)	0.844	0.0275<br>(0.074)	0.710	0.00028<br>(0.0003)	0.407	-0.0082<br>(0.0185)	0.658	**2.06e-07<br>(5.55e-08)**	**0.001**
Plasmablasts	0.0004<br>(0.0007)	0.595	-0.029<br>(0.029)	0.325	0.0003<br>(0.111)	0.998	-0.0003<br>(0.0005)	0.599	-0.037<br>(0.033)	0.261	**5.38e-07<br>(8.96e-08)**	**0.000**
**Infants' memory B cell recall responses**												
PCPs	-0.0006<br>(0.0013)	0.658	-0.150<br>(0.122)	0.232	0.354<br>(0.488)	0.473	0.001<br>(0.0035)	0.772	.0041825<br>.084301	0.961	-3.82e-07<br>1.31e-06	0.773
Measles	-0.0009<br>(0.005)	0.868	-0.127<br>(0.201)	0.535	0.223<br>(0.629)	0.721	0.0015<br>(0.0039)	0.702	-.054768<br>.1267134	0.670	1.61e-06<br>1.86e-06	0.396
TT	0.0034<br>(0.0041)	0.414	-0.025<br>(0.171)	0.887	0.443<br>(0.843)	0.603	0.003<br>(0.0038)	0.370	-.0327105<br>.1291981	0.802	1.10e-06<br>1.03e-06	0.296
Diphtheria	0.0004<br>(0.0025)	0.875	0.111<br>(0.086)	0.209	-0.026<br>(0.601)	0.966	0.002<br>(0.0027)	0.373	1102837<br>.0486993	0.031	3.51e-07<br>7.64e-07	0.650
Total IgG	0.047<br>(0.085)	0.585	1.555<br>(3.43)	0.654	-3.890<br>(13.51)	0.775	0.0311<br>(0.0565)	0.586	2.886192<br>3.688366	0.440	-9.64e-06<br>.0000182	0.601
**Infants' antigen specific antibody levels**												
PCPs	-0.0005<br>(0.0006)	0.366	-0.0177<br>(0.033)	0.598	-0.139<br>(0.139)	0.321	-0.0002<br>(0.0005)	0.664	-0.040<br>(0.043)	0.359	5.63e-07<br>5.46e-07	0.308
Measles	-0.001<br>(0.0008)	0.227	-0.0311<br>(0.0422)	0.464	-0.0148<br>(0.177)	0.934	-0.0006<br>(0.0007)	0.373	-0.049<br>(0.055)	0.372	-1.63e-07<br>7.05e-07	0.819
TT	-0.0004<br>(0.0007)	0.618	-0.056<br>(0.040)	0.167	-0.274<br>(0.167)	0.108	0.00016<br>(0.0007)	0.815	-0.043<br>(0.052)	0.415	3.80e-08<br>6.68e-07	0.955
Diphtheria	-0.0014<br>(0.00096)	0.149	-0.009<br>(0.049)	0.848	0.080<br>(0.204)	0.699	-0.00092<br>(0.0009)	0.339	-0.011<br>(0.064)	0.869	9.71e-07<br>7.20e-07	0.186
HiB	-0.0008<br>(0.0014)	0.561	-0.071<br>(0.065)	0.291	0.0219<br>(0.307)	0.944	0.0007<br>(0.0014)	0.637	-0.0179<br>(0.094)	0.094	-1.58e-06<br>7.70e-07	0.054
RSV	0.0002<br>(0.0014)	0.914	0.061<br>(0.066)	0.362	0.226<br>(0.285)	0.435	-0.00029<br>(0.0013)	0.818	0.183<br>(0.086)	0.042	-1.27e-07<br>9.50e-07	0.894
Total IgG	-0.0002<br>(0.0007)	0.789	-0.0006<br>(0.036)	0.986	0.186<br>(0.149)	0.219	0.0003<br>(0.00061)	0.593	0.0019<br>(0.046)	0.967	-1.52e-07<br>5.91e-07	0.798

Linear regression models were used to describe the association between maternal parameters taken within four months after the infant's birth (CD4 counts, body mass

index (BMI) and duration on Highly active antiretroviral therapy (HAART)) and at recruitment (CD4 counts, BMI and viral load), with the infant’s immunological outcomes. The Beta co-efficient and standard error are indicated. P values less than 0.05 were considered significant and indicated in bold. MBC; memory B cells, PCPs; pneumococcal capsular polysaccharides, TT; Tetanus toxoid, HiB; haemophilus influenzae type b, RSV; Respiratory Syncytial virus
